# Progress in Surgery

**Published:** 1897-03-27

**Authors:** 


### Progress in Surgery.

### SURGERY OF THE KIDNEYS AND URETERS.

**The Diagnosis of Hæmaturia** is not always easy, but Morris[1] points out that hæmaturia of vesical can be diagnosed from that of renal origin, in some cases, by bimanual examination. By placing the index finger of one hand in the rectum whilst the other hand forces the empty bladder into the pelvis by firm pressure upon the abdominal wall immediately above the pubes, he has succeeded in demonstrating the presence of a calculus in cases where it was not before suspected. The method is necessarily of greater value in the female than in the male.

**Tuberculosis of the Kidney** is thus summed up by Holmes[2]: (1) It is a relatively common disease. (2) It usually begins in the kidney itself, descends through the ureter to the bladder, and ascends to the opposite kidney. (3) It is, therefore, for a long time a unilateral disease. (4) It is progressive and destructive, not subject to improvement by medication, offering an unfavourable prognosis as to life and comfort, and subject to extension downward by the urinary tract, and outward through the peri-renal lymphatics. (5) Diagnosis can be made by the symptoms of cystitis, with low temperature, rapid pulse, and dilatation of the heart; the detection of tubercle bacilli in the urine; tuberculosis of the bladder about the orifice of the ureter of the diseased kidney; pus or blood, with tubercle bacilli and diminished normal constituents in the urine from the diseased kidney; normal urine in increased quantity from the opposite kidney; sometimes tenderness, pain, and tumour *in situ* of diseased kidney and ureter. (6) The indications, in case of an absolute diagnosis of tuberculosis of one kidney and healthy opposite kidney, are immediate removal of diseased kidney and its ureter; in case of disease in both kidneys, no operation should be performed. (7) The competency of the healthy kidney should be proved by repeated catheterisation of the ureters before nephrectomy, and the removal of all toxic elements from the blood should be secured by a liquid diet, irrigation of the colon, and hydration of the whole system for some days before the removal of the kidney. (8) Lumbar extra-peritoneal nephrectomy is the safer operation. (9) In women the removal of the ureter should be completed through the vagina. (10) Any remaining tuberculosis of the bladder should be treated locally by curetting or cauterisation. (11) Catheterisation of the ureter is not a dangerous operation, and it may be easily accomplished in women by the cystoscopes of Simon, Pawlik, or Kelly, and in men with the more complicated instrument of Casper.

**Movable Kidney.**—Cordier[3] has made the following deductions: (1) This condition often produces a dilatation of the stomach with all the accompanying symptoms of a disease of that organ. (2) It is a fruitful source of gall-stones, because of the pedicle producing a partial obstruction of the common duct. (3) The bending of the ureter often gives rise to a hydronephrosis, which in turn may be converted into a pyonephrosis. (4) It may produce death by a complete strangulation from torsion of the vessels and ureter. (5) By dragging on the abdominal aorta and kinking the vena cava, a condition simulating an aneurism may be produced. (6) Pain of a referred character to the region of distribution of the spinal nerves is often induced. (7) Neurasthenia may be caused by the interference of this condition with digestion, assimilation, and elimination. (8) Nephrorraphy is a safe and effective surgical procedure. (9) All cases of movable kidney, if accompanied by symptoms pointing to the kidney as their source, should be operated on. (10) Symptoms are not to be relied upon in making a diagnosis. Physical examination is the only trustworthy guide. Taylor[4] classifies nephroptosis under three clinical heads: (1) Patients who have displaced kidney, do not know it, and suffer no inconvenience from it; this type, he thinks, represents by far the largest class. (2) Patients with displaced kidney, who may or may not know it, who suffer from gastroenteric discomfort, and perhaps a long train of vague neurotic disturbances. In this type we find the largest class calling for operative interference. (3) Patients with movable kidney who are subjects of occasional or frequent mild or severe attacks of renal crises. This is the least frequent type met with, but the urgency of the symptoms more frequently demands operative interference. He believes that the Deitl's or renal crisis is due to a kink or twist of the ureter, with retained urine in the ureter and pelvis of the kidney. The tendency of this condition to produce hydronephrosis or pyonephrosis renders operation more imperative in these cases.

**Traumatic Lesions of the Kidney.**—Speaking of wounds other than those caused by gun-shot, Keen[5] says: (1) The usual external disinfection should be scrupulously carried out. (2) Both for examination and for arrest of hæmorrhage the primary wound should be enlarged, if necessary, and disinfected. (3) All hæmorrhage should be arrested as quickly as possible. (4) If the kidney is not wounded, a temporary drain may be placed in the wound, and the greater part of it closed. (5) If the kidney is wounded also, this wound should be sutured or a partial nephrectomy be done, followed by drainage and partial closure of the wound. (6) If the pelvis be opened, it should be sutured if possible, and the wound drained. (7) If the large vessels are cut, or the kidney itself is lacerated beyond the point at which it would probably retain its vitality and recover its function, immediate nephrectomy should be done. (8) If, in spite of disinfection, as a result of the primary wound, perinephric abscess should follow, this should be widely opened and thoroughly drained, and if the kidney be hopelessly involved, or the condition of the patient so serious as to demand it, secondary nephrectomy should be done. (9) If the peritoneal cavity has been entered by the vulnerating body, carrying infection in its course, and all the more if the viscera be involved, abdominal section should be done at once, the kidney treated as above indicated, and other lesions remedied. Interesting cases bearing on these points are recorded by Keen,[6] Wallis,[7] and Richardson.[8]

**Cystonephrosis**, according to Fenger,[9] should be treated on conservative lines, and demands a number of successive operations: I. Hydronephrosis.—(1) Lumbar nephrotomy, followed by packing and aseptic drainage. (2) If an urinary fistula remains after three months an operation is necessary for the stenosis, namely, for (*a*) stricture of ureter, or (*b*) valve formation and oblique insertion. (3) If the fistula still remains with the ureter patent, which occurs only when there is obstruction above the ureter, the kidney should be bisected. When the entire territory of the sac is thus laid open and the ureter is patent, as demonstrated by free passage of bougies from the kidney to the bladder, and by free passage of injected fluid, then (4) closure of the fistula by reunion of the bisected kidney. It should not, however, be done until the pyelitis, if present, has been cured by thorough irrigation from the kidney to the bladder. II. Pyonephrosis—(1) Lumbar nephrotomy, followed by drainage and local treatment of the pyelonephritis. (2) When the fistula persists, when considerable healthy urine passes through it, and the kidney has contracted to a reasonable degree—that is, in two or three months—search must be made for the obstruction, and an operation undertaken for its relief: (*a*) Stricture of ureter and valve formation at its pelvic entrance. If this condition, with an ureter patent from pelvis to bladder, still does not prevent the passage of large quantities of urine through the lumbar fistula, obstruction above the pelvis should be suspected, and (*b*) operation as for sacculated kidney, with bisection and division of the partition walls of the calyces and of the branches of the ureter. When the whole territory of secreting kidney tissue is thus in unimpeded connection with the bladder, and still spontaneous closure does not take place, although the pyelitis is almost cured, the final step to effect closure of the fistula should be taken, namely (3), closure of the bisected kidney, by loosening the adhesions of its two halves to the abdominal wall, and by suturing the kidney substance together. Cases of pyonephrosis are reported by Myles[10] and Nash.[11]

[1] Lancet, Oct. 31st, 1896. [2] New York Med. Journ., Nov. 14th, 1896. [3] Med. Record, New York, Oct. 10th, 1896, p. 527. [4] Ibidem, p. 527. [5] Annals of Surgery, Aug., 1896. [6] Loc. cit. [7] Brit. Med. Journ., Oct. 31st, 1896. [8] Annals of Surgery, Dec., 1896. [9] Ibidem, Nov., 1896. [10] Brit. Med. Journ., Nov. 21st, 1896. [11] Ibidem, Nov. 14th, 1896.

### SURGERY OF THE NERVES.

**Trifacial Neuralgia.**—In a recent number of The Hospital (October, 1896) a valuable paper by Dr. Giles de la Tourette on the medical treatment of trifacial neuralgia was reviewed. Since then a considerable amount has been written on the treatment of this most distressing condition from the surgical point of view.

The less severe forms of operation, such as stretching, section, and resection of a portion of the affected nerve which have for long been relied upon, are gradually giving place to much more extensive and grave procedures. The minor operations have proved in practice to grant but slight benefit in some cases, and only temporary relief in most. Simple section of a nerve is soon followed by reunion, and with this the pain returns, in many cases with astonishing rapidity.

**Nerve Section.**—Corning[1] believes that if this re-establishment of the continuity of the nerve could be prevented good results might be expected from the subcutaneous division of offending nerves. He has devised a method by which the nerve is subcutaneously severed with a small, broad, sickle-shaped, and hollow knife, and the ends forcibly pushed apart by lateral pressure with the knife blade. Into the space thus formed a quantity of a non-irritant and aseptic oil, with a melting point from 3 to 5 deg. above that of the blood, is introduced by means of a syringe, which forms the handle of his hollow-bladed knife. The mixture injected consists of oil of theobroma, with the addition of a sufficient quantity of paraffin to bring the melting point up to about 105 deg. Fahr. A quantity of this mixture is injected into the region of the affected nerve, and congealed by the external application of ice, before the hollow-bladed knife is introduced for the section of the nerve. No untoward results have followed the presence of this mixture in the tissues. Cases of cervico-occipital neuralgia are most suitable for the method, but it is available for other nerves as well. Two cases treated in this way are reported in his paper, but in both the freedom from pain has only lasted "several weeks" at the date of writing.

**Nerve Stretching.**—Dr. West[2] reports a typical case of tic doloureux in a female, aged 33, successfully treated by nerve stretching after all the ordinary means of treatment by drugs had signally failed. The infra-orbital branch of the fifth nerve was at fault, and for 13 months, save for a few slight twinges of pain at long intervals, the patient has been in perfect comfort, and her general health has greatly improved.

**Thiersch's Method of Nerve Avulsion.**—A more radical local operation than that of nerve stretching is the avulsion method of Thiersch, in which the nerve is exposed and a considerable portion of the trunk, with its ramifications, forcibly extracted by slow traction with forceps. Angerer, of Munich,[3] has performed this operation on 26 patients since 1889, 52 nerves in all having been operated upon. The supra-orbital was attacked 14 times, the infra-orbital 16 times, the mental branch nine times, the infra-maxillary nerve seven times, and the lingual branch once. At the time of reporting 17 out of the 26 patients were free of pain, the immunity having lasted in some cases as long as four years. Taking the cases operated on since June, 1892—16 in number—three will require further operation; in other three relapses occurred; one patient died six months after the operation from an intercurrent affection; two were distinctly improved; and seven remained free from pain. In these seven patients 16 nerves were avulsed.

Angerer commends this method on the grounds of its results, its safety, the avoidance of a disfiguring scar, and the chances it offers of avoiding the necessity for the much more serious operations on the Gasserian ganglion. The associated anæsthesia is never seriously complained of by the patients. Hefferich, of Grifeswald (ibid.) confirms the observations of Angerer; while Krause has not obtained such satisfactory results. He has seen in his own practice, and in that of others, about half the cases suffer from recurrence of pain. He suggests that resection is probably as good as avulsion.

Thomson (Edinburgh)[4] completely relieved a patient of neuralgia of the second division of the fifth by section and removal of portion of the nerve close up to the foramen rotundum, after Kocher's method. A considerable amount of deformity ensued.

**Intra-Cranial Operations.**—In a valuable paper[5] L. M. Tiffany, of Baltimore, records the results of his own experience, and of a study of 108 cases which he has collected of intra-cranial operations for the relief of trifacial neuralgia. While admitting that peripheral operations cure many cases, some do recur, and he is led to the conclusion that our best hope is in attacking the Gasserian ganglion. He is careful to remark that undoubtedly some cases of this affection are due to a central (cerebral) cause; and he suggests that investigation will some day reveal a cerebral area, excision of which will afford relief to those cases where removal of the ganglion of Gasser fails.

After referring to the various operations which have been carried out with a view to reaching this ganglion, he specially details the steps of Doyen's operation, a description of which, with illustrations, will be found in The Hospital of May 2nd, 1896.

The majority of operators seem to prefer the Hartley-Krause operation. Out of the 108 cases collected by Tiffany, nearly two-thirds were subjected to the Hartley-Krause procedure; nearly one-fourth to that of Rose; seven to that of Horsley; to that of Doyen, four; Quénu, four; Novaro, one; and in one other no method is mentioned.

The patients varied in age from 21 to 79, and males were slightly in excess of females; race appears to have no influence in the production of neuralgia.

The right side of the face was affected twice as often as the left. The first division of the nerve was never involved alone; it appears always to be affected reflexly if at all. In the majority of the cases operated upon the pain involved the second and third branches together. Seldom can a satisfactory cause be discovered, although the patients usually attribute the onset to some definite event. In one exceptional case an exostosis near the foramen ovale satisfactorily explained the pain. The only constant pathological condition found in those suffering from this disease is arterio-sclerosis, and this exists both in the nerve trunks and ganglia in many cases operated upon.

Tiffany agrees with the majority in preferring the Hartley-Krause method of operating, and details the steps of the operation. He (Tiffany) does not now replace the bone-flap. He finds the raising of the brain greatly facilitated by permitting the escape of cerebro-spinal fluid through an opening in the dura. He prefers to leave the upper third of the ganglion with the first division of the nerve, for fear of interfering with the trophic supply of the eye. The motor root should be preserved if possible. It is highly advantageous to complete the operation at one sitting, and to afford drainage to the wound.

Shock and sepsis are the two great risks of the operation. When the first division is removed trophic eye disturbances may follow. The mortality over all the 108 cases collected is about 22 per cent. The results as regards pain are encouraging. In all cases temporary relief is afforded, and in most it is permanent. Recurrence of pain after complete and certain removal of the ganglion of Gasser is not recorded.

Tiffany sums up the indications for the operation thus: Intracranial operation is to be thought of (*a*) if more than one branch is affected; (*b*) if the painful area receives filaments from the branches near the exit from the head, *e.g.*, tongue, temporal region; (*c*) if the pain is not the expression of a constitutional disease; (*d*) if a cause central to the ganglion does not exist; (*e*) if other measures have failed to relieve.

Murphy[6] contributes a paper on the same lines as that of Tiffany, and his conclusions, based on his own experience, are practically the same. He has not met with ocular symptoms in his own cases after operation.

Barclay[7] refers to Turner and Ferrier's investigations on the trophic influence exerted by the Gasserian ganglion on the cornea, which go to show that, provided septic organisms be excluded, the ophthalmic branch or the Gasserian ganglion may be removed without fear of disorganisation of the eye. Krause's clinical experience goes to support this opinion. Gaule's experiments on the same point lead to quite opposite conclusions.

Poirier[8] considers the intra-cranial operation useful and comparatively easy. After dividing the second and third divisions of the nerve at the foramen ovate and rotundum, he seizes the ganglion, and by forcible traction on it avulses the nerve trunk from the medulla.

[1] New York Med. Rec., Sep. 5, 1896. [2] Brit. Med. Jour., Jan. 9, 1897. [3] Centralbl. f. Chirurg., Aug. 1896. [4] Edin. Med. Jour., Oct., 1896. [5] Arm. of Surgery, Dec., 1896. [6] Amer. Med.-Surg. Bulletin, Oct., 1896. [7] Bristol Med.-Chir. Jour., March, 1896. [8] Medical Week, July, 1896.

